# DPP6 and MFAP5 are associated with immune infiltration as diagnostic biomarkers in distinguishing uterine leiomyosarcoma from leiomyoma

**DOI:** 10.3389/fonc.2022.1084192

**Published:** 2022-11-30

**Authors:** Yumin Ke, LiuXia You, YanJuan Xu, Dandan Wu, Qiuya Lin, Zhuna Wu

**Affiliations:** ^1^ Department of Gynecology and Obstetrics, The Second Affiliated Hospital of Fujian Medical University, Quanzhou, Fujian, China; ^2^ Department of Clinical Laboratory, The Second Affiliated Hospital of Fujian Medical University, Quanzhou, Fujian, China; ^3^ Department of Pathology, The Second Affiliated Hospital of Fujian Medical University, Quanzhou, Fujian, China; ^4^ Department of Gynecology, The First Hospital of Quanzhou Affiliated to Fujian Medical University, Quanzhou, Fujian, China

**Keywords:** diagnostic biomarkers, machine-learning, DPP6, MFAP5, immune infiltration, uterine leiomyosarcoma

## Abstract

**Objective:**

Uterine leiomyosarcoma (ULMS) is the most common subtype of uterine sarcoma and is difficult to discern from uterine leiomyoma (ULM) preoperatively. The aim of the study was to determine the potential and significance of immune*-*related diagnostic biomarkers in distinguishing ULMS from ULM.

**Methods:**

Two public gene expression profiles (GSE36610 and GSE64763) from the GEO datasets containing ULMS and ULM samples were downloaded. Differentially expressed genes (DEGs) were selected and determined among 37 ULMS and 25 ULM control samples. The DEGs were used for Gene Ontology (GO), Kyoto Encyclopaedia of Genes and Genomes (KEGG) and Disease Ontology (DO) enrichment analyses as well as gene set enrichment analysis (GSEA). The candidate biomarkers were identified by least absolute shrinkage and selection operator (LASSO) and support vector machine recursive feature elimination (SVM-RFE) analyses. The receiver operating characteristic curve (ROC) was applied to evaluate diagnostic ability. For further confirmation, the biomarker expression levels and diagnostic value in ULMS were verified in the GSE9511 and GSE68295 datasets (12 ULMS and 10 ULM), and validated by immunohistochemistry (IHC). The CIBERSORT algorithm was used to calculate the compositional patterns of 22 types of immune cells in ULMS.

**Result:**

In total, 55 DEGs were recognized *via* GO analysis, and KEGG analyses revealed that the DEGs were enriched in nuclear division, and cell cycle. The recognized DEGs were primarily implicated in non−small cell lung carcinoma and breast carcinoma. Gene sets related to the cell cycle and DNA replication were activated in ULMS. *DPP6* and *MFAP5* were distinguished as diagnostic biomarkers of ULMS (AUC = 0.957, AUC = 0.899, respectively), and they were verified in the GSE9511 and GSE68295 datasets (AUC = 0.983, AUC = 0.942, respectively). The low expression of *DPP6* and *MFAP5* were associated with ULMS. In addition, the analysis of the immune microenvironment indicated that resting mast cells were positively correlated with *DPP6* and *MFAP5* expression and that eosinophils and M0 macrophages were negatively correlated with *DPP6* expression (P<0.05).

**Conclusion:**

These findings indicated that *DPP6* and *MFAP5* are diagnostic biomarkers of ULMS, thereby offering a novel perspective for future studies on the occurrence, function and molecular mechanisms of ULMS.

## Introduction

Uterine leiomyosarcoma (ULMS) is a rare but aggressive tumor subtype, accounting for approximately 1% of all uterine malignancies (1). ULMS is the most common subtype of uterine sarcoma and originates from the smooth muscles of the myometrium. In the past several decades, the prognosis of ULMS patients has not changed with an overall *5*-year survival rate of only 15%‐25% (2). Currently, complete surgical resection is the primary treatment for early-stage ULMS (3), and chemotherapy is regarded as the standard therapy for advanced or metastatic ULMS (4, 5), but with an estimated recurrence* *rate of* *approximately 50* *to* *70% (6). ULMS constitutes a* *sizable proportion* *of uterine cancer deaths (7). Additionally, compared to other gynecological malignancies, ULMS etiology, pathogenesis and earlier diagnosis are poorly understood. Considering that ULM can currently be treated with minimally invasive surgery, it is important to discern ULMS from ULM preoperatively to avoid disseminated spread by laparoscopic morcellation or delayed diagnosis with conservative treatment (8, 9). Considering that ULMS has a high trend towards local recurrence, metastasis and poor prognosis, the misdiagnosis of a ULMS for a leiomyoma may lead to therapy delays and higher morbidity (10, 11).

ULMS patients generally present with abnormal vaginal bleeding, pelvic pain and palpable pelvic mass. Because these symptoms resemble ULM, particularly degenerated ULM, it is difficult to discern ULMS and ULM by pelvic ultrasound and MRI preoperatively (12). Postoperative pathological diagnosis is currently the only available method to distinguish the two tumor conditions. A meta-analysis containing 133 studies has indicated that undiagnosed ULMS estimated to be approximately 1 in 2000 surgeries for presumed ULM (13). It* *is* *well known* *that* *tumor-associated immunity* *plays a vital role in the occurrence, development and metastasis of tumors (14). The recent development of integrated microarray technology with bioinformatics* *analysis may allow identification of novel genes that might act as diagnostic and prognostic biomarkers in cancers (15, 16). Definitive molecular diagnosis added to histopathological diagnosis should be considered to decrease the risk of misdiagnosis. Verification of highly novel diagnostic biomarkers for ULMS related to immune cell infiltration will further improve the diagnostic accuracy of ULMS.

Herein, the aim of this study was to identify novel diagnostic immune-related genes for ULMS. Machine-learning algorithms and logistic regression were used to verify diagnostic biomarkers of ULMS. Furthermore, CIBERSORT was applied to compute the quotas of infiltrating immune cells between ULMS and ULM samples. Finally, the correlation among the recognized diagnostic biomarkers and infiltrating immune cells was explored to offer a foundation for further research.

## Materials and methods

### Microarray data processing and identification of DEGs

First, we obtained datasets (GSE36610 and GSE64763 as the training group; and GSE9511 and GSE68295 as the testing group) from the GEO database (https://www.ncbi.nlm.nih.gov/gds) (**Table 1**). The background correction and normalization of raw data were processed by the limma package of R software (http://www.bioconductor.org/). Two datasets were merged into a metadata cohort, and the batch effect was removed with the SVA package of R software (17). Genes with |log fold change (FC)| > 2 and adjusted* *P *<* 0.05 were defined as DEGs.

**Table 1:** GEO database data of preeclampsia mRNA expression profile.

Dataset ID	Platform	leiomyosarcoma	leiomyoma
Train group			
GSE36610	GPL7363-11635	12	0
GSE64763	GPL571-17391	25	25
Test group			
GSE9511	GPL80-30376	9	7
GSE68295	GPL6480-9577	3	3

### Functional enrichment of DEGs

The DEGs were analyzed using the clusterProfiler, org.Hs.eg.db, enrichplot and ggplot2 packages of R software for GO and KEGG analyses. The clusterProfiler and DOSE packages of R software were used to perform DO analyses on DEGs. GSEA was conducted to recognize the most important feature between the ULMS and ULM groups. “c2.cp.kegg.v7.4.symbols.gmt” was applied as the reference gene set from the Molecular Signatures Database (MSigDB). P <0.05 was considered a significant enrichment.

### Screening candidate biomarker for diagnosis of ULMS

We applied two machine-learning algorithms to increase the prediction* *accuracy. LASSO* *is a regression-based analysis that scrutinizes variable selection and regularization in ULMS models. The glmnet package of R software was applied to perform LASSO regression analysis on the identification of DEGs correlated with the discernment between ULMS and ULM. The support vector machine (SVM) is an efficient and widely applied supervised machine-learning algorithm for disease classification and regression tasks (18). Consequently, we screened the overlapping genes by conjugating LASSO and SVM-RFE followed by verification using the GSE9511 and GSE68295 datasets.

### Significance of diagnostic biomarkers in ULMS

We obtained mRNA expression data from 37 ULMS and 25 ULM samples, which were applied to create ROC curves to verify* *the biomarker predictive ability. The area under the ROC curve (AUC) was utilized to determine the ability of diagnosis in distinguishing ULMS from ULM samples followed by verification using the GSE9511 and GSE68295 datasets.

### Evaluating the level of immune infiltration

We downloaded a gene signature matrix with interpretation, known as the 22 immune cell (LM22) matrix with 1,000 permutations from CIBERSORT (http://cibersort.stanford.edu/) (19). The CIBERSORT algorithm was applied to quantify the proportion of 22 infiltrating immune cells in the tissue using the expression of 547 immune-related genes. The corrplot package in R software was applied to conduct the correlation and visualization of 22 types of infiltrating immune cells. The vioplot package in R software was used to study the infiltration of immune cells between the ULMS and ULM groups. Pearson correlation analysis was applied to explore the selected diagnostic biomarkers correlated with the levels of infiltrating immune cells.

### Patient and tissue samples

Twenty-six paraffin-embedded ULMS and twenty-three ULM specimens were diagnosed at The Second Affiliated Hospital of Fujian Medical University (Fujian, China) from September 2010 to February 2022. The main treatment of all patients underwent hysterectomy with bilateral adnexal resection. The research was approved by the Research Ethics Committee of The Second Affiliated Hospital of Fujian Medical University prior to the study.

### Immunohistochemistry

IHC staining was operated as previously described (20). The primary antibodies included anti-DPP6 (Bioss, Beijing), anti-MFAP5 (Proteintech, USA). The proportion of DPP6 and MFAP5 staining intensity was scored as follows: negative = 0; light yellow = 1; brownish yellow = 2; or tan=3. The staining was scored as follows: less than 1/3 = 1; between 1/3 and 2/3 = 2; or more than 2/3 = 3. The final score for DPP6 and MFAP5 expression was calculated by multiplying the 2 scores. The slides were classified to low and high expression group, corresponding to scores of <3 or ≥3, respectively. The histopathological diagnosis of the patients included in our study was established by two pathologists specialized in Gynecologic Oncology.

### Statistical analysis

We utilized R software to conduct (v.4.1.1) all statistical analyses. We used the Mann–Whitney U test to compare the different groups. LASSO regression, SVM algorithm, ROC curve, Pearson’s correlation and unpaired t test were used as described above. Differences with P < 0.05 were considered statistically significant for all statistical analyses.

## Result

### Study procedure

**Figure 1:**
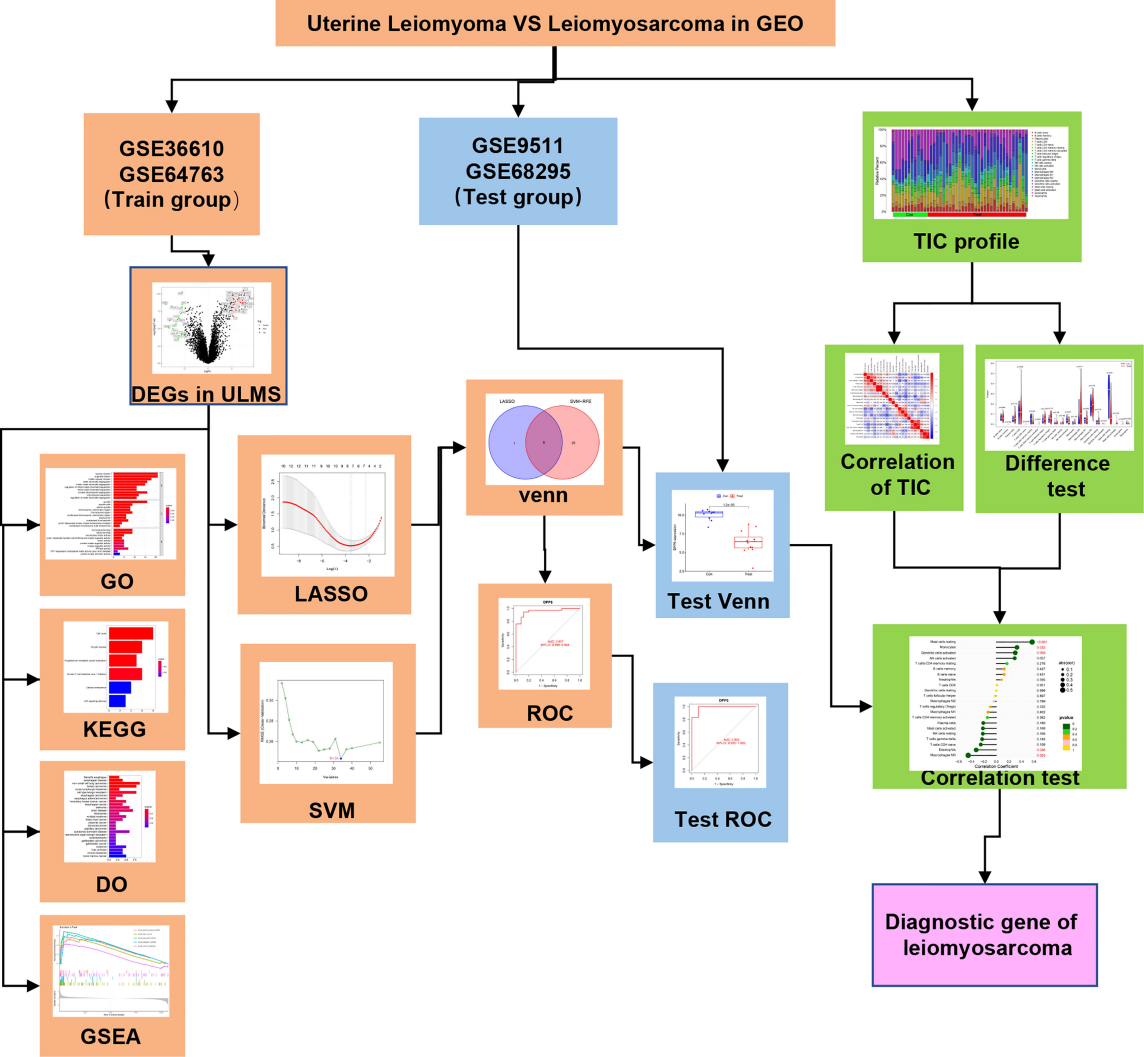
Analysis flow diagram of this study.

The analysis procedure of the present is shown in **Figure 1**. The transcriptome RNA-seq data were downloaded from the GEO database. We identified the DEGs between the ULMS and ULM group. DEGs were anlayzed using the GO, KEGG and DO analyses as well as GSEA. LASSO and SVM-RFE were used to select the candidate overlapping genes, and ROC curves were applied to* *check the* *predictive* *ability of biomarkers, which was further verified in the GSE9511 and GSE68295 datasets. The compositional patterns of 22 immune cells were calculated using CIBERSORT in ULMS. Finally, correlation analysis among the diagnostic markers and infiltrating immune cells was performed.

### Identification of DEGs in ULMS

**Figure 2:**
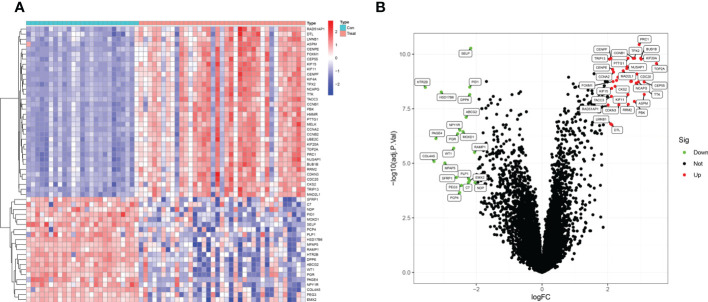
Identifcation of DEGs. **(A)** Heatmap plots of 55 DEGs between ULMS and ULM samples from GEO database. Row name of heatmap is the gene name, and column name is the ID of samples which not shown in plot.The colors from red toblue represent expression level from high to low in the heatmaps. **(B)** Volcano plots of top 50 DEGs between ULMS and ULM samples. The red dots in the volcano plots represent up-regulation, the green dots represent down-regulation and black dots represent genes without differential expression.

The present study utilized two datasets (GSE36610, GSE64763) and included 37 ULMS and 25 ULM samples. We identified 55 DEGs by comparing ULMS and ULM (**Figure 2A**). Among* *these DEGs, 21 genes were significantly downregulated, and 34 genes were significantly upregulated. The volcano plot in **Figure 2B** shows the distribution of the top 50 DEGs in ULMS and ULM.

### Correlation and functional enrichment analysis

**Figure 3:**
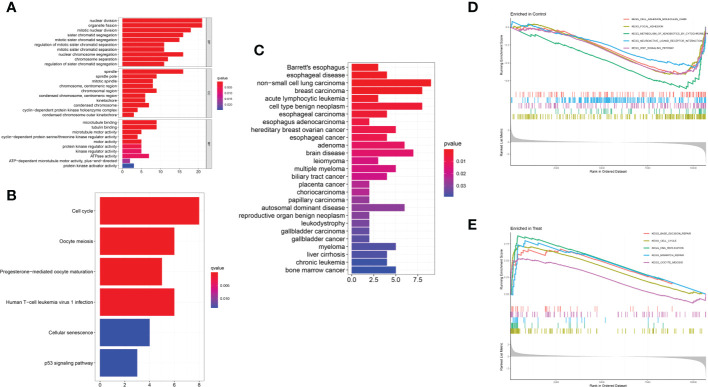
Functional enrichment analyses to identify potential biological processes. **(A)** GO analysis. GO analysis divided DEGs into three functional groups: molecular function (MF), biological processes (BP), and cell composition (CC). **(B)** KEGG analysis of DEGs. **(C)** Disease ontology enrichment analysis of DEGs between ULMS and ULM samples. **(D, E)** Enrichment analyses between ULMS and ULM samples *via* gene set enrichment analysis.

The GO analysis indicated that the DEGs mainly participated in chromosome segregation and the cell cycle (**Figure 3A**). In addition, KEGG analysis showed enrichment of the cell cycle and immune-related pathways, such as HTLV-1 infection way (**Figure 3B**). The DO enrichment showed that DEGs were mostly related to solid malignant tumors and haematological malignancies (**Figure 3C**). The GSEA* *results revealed negative enrichment in cell adhesion and the Wnt signalling pathway in ULM (**Figure 3D**, **Table S1**) as well as positive enrichment in the cell cycle, DNA replication and mismatch repair in ULMS (**Figure 3E**, **Table S2**). These results indicated that mismatch repair, related immunity and the cell cycle play vital roles in ULMS.

### Verification and validation of diagnostic biomarkers

**Figure 4:**
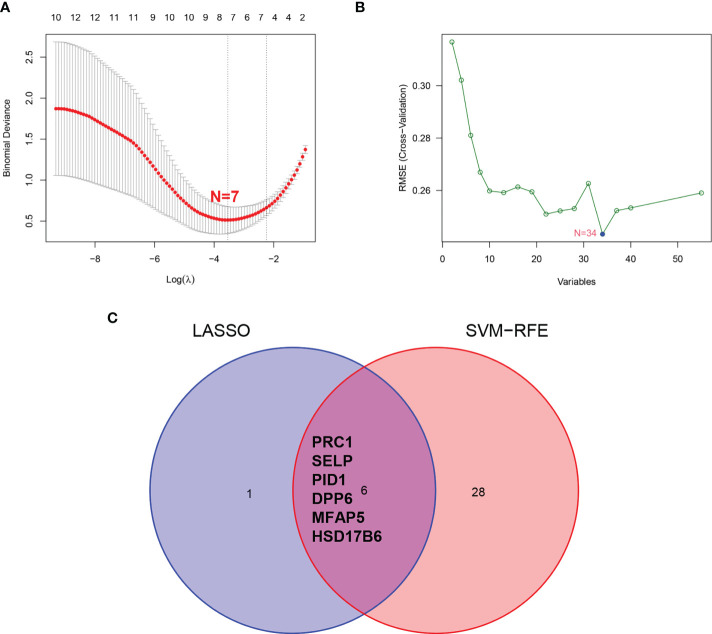
Screening process of diagnostic biomarker candidates for ULMS diagnosis. **(A)** Tuning feature selection in the LASSO model. **(B)** A plot of biomarkers selection *via* SVM-RFE algorithm. **(C)** Venn diagram demonstrating 6 diagnostic biomarkers shared by the LASSO and SVM-RFE algorithms.

**Figure 5:**
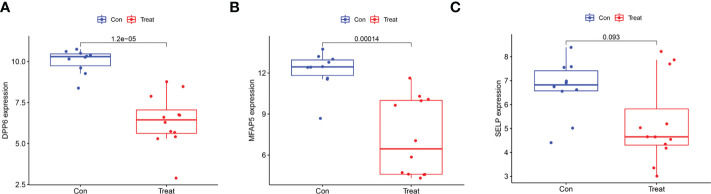
Validation of the expression of diagnostic biomarkers in the GSE9511 and GSE68295. **(A)** *DPP6*; **(B)** *MFAP5*; **(C)** *SLEP*.

We employed the LASSO and SVM-RFE algorithm methods to select potential biomarkers. We identified 7 DEGs as diagnostic biomarkers using LASSO regression for ULMS (**Figure 4A**), and we verified 34 DEGs using the SVM-RFE (**Figure 4B**). When integrating both algorithms, six overlapping candidate genes (*PRC1*, *SELP*, *PID1*, *DPP6*, *MFAP5* and *HSD17B6*) were selected (**Figure 4C**). In addition, with the purpose of producing more dependable and exact DEGs, we verified the expression levels of six DEGs using the GSE9511 and GSE68295 datasets. The *DPP6* and *MFAP5* expression levels in ULMS samples were significantly lower than those in the ULM group (**Figures 5A, B**; P < 0.05). However, *SLEP* gene expression was not significantly different between the two groups (**Figure 5C**). Subsequently, we investigated the latent ability of the two identified DEGs as diagnostic biomarkers utilizing a logistic* *regression algorithm.

### Effectiveness of diagnostic biomarkers in ULMS

**Figure 6:**
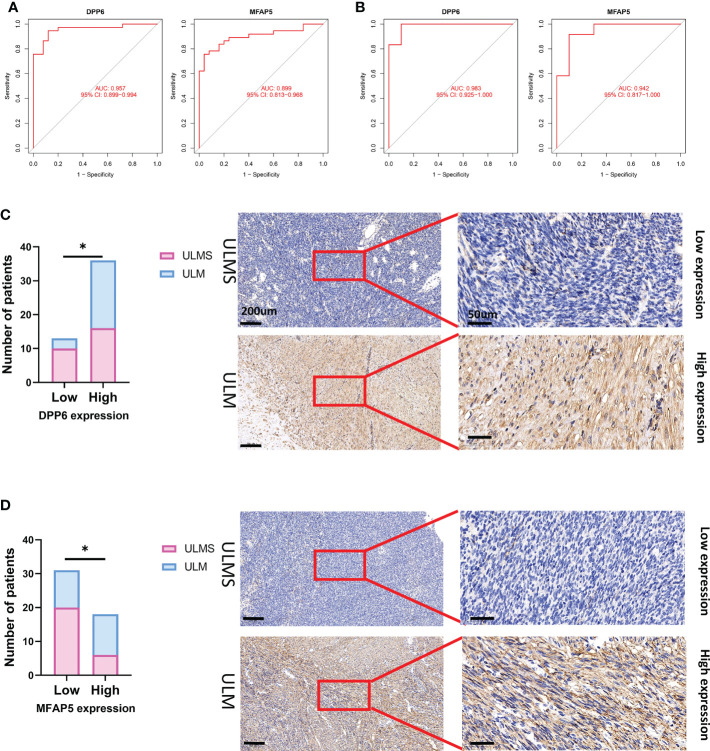
The receiver operating characteristic (ROC) curve of the diagnostic effectiveness of the six diagnostic markers. **(A)** ROC curve of *DPP6* and *MFAP5* after fitting to one variable in the metadata cohort; **(B)** ROC curve of *DPP6* and *MFAP5* after fitting to one variable in the GSE9511 and GSE68295 dataset. **(C)** Significantly low *DPP6* expression was observed in ULMS tissues compared with ULM specimens (ULMS=26, ULM=23). Representative images (×50 and ×400) of IHC staining for *DPP6* in 26 ULMS and 23 ULM patients (high expression vs. low expression). **(D)** Significantly low *MFAP5* expression was observed in ULMS tissues compared with ULM specimens (ULMS=26, ULM=23). Representative images (×50 and ×400) of IHC staining for *MFAP5* in 26 ULMS and 23 ULM patients (high expression vs. low expression). Scale bars are shown. *P < 0.05. P values were calculated by chi-square tests.

The ability of the two diagnostic biomarkers indicated good diagnostic value in early discernment of ULMS as the AUC values of the *DPP6* and *MFAP5* genes were 0.957 and 0.899, respectively (**Figure 6A**). Subsequently, a persuasive screening capacity was verified in the GSE9511 and GSE68295 datasets with AUC values of 0.983 in *DPP6* and 0.942 in *MFAP5* (**Figure 6B**). We assessed the expression of *DPP6* and *MFAP5* across ULMS and ULM tissues *via* immunohistochemistry and found that low expression of *DPP6* and *MFAP5* were associated with ULMS. DPP6 was expressed in the cytoplasm, MFAP5 was expressed in the stroma. (**Figures 6C, D**; P < 0.05). The above results indicating that the *DPP6* gene and *MFAP5* had a higher diagnostic capacity.

### *DPP6* and *MFAP5* genes correlate with the percentage of immune cell infiltration

**Figure 7:**
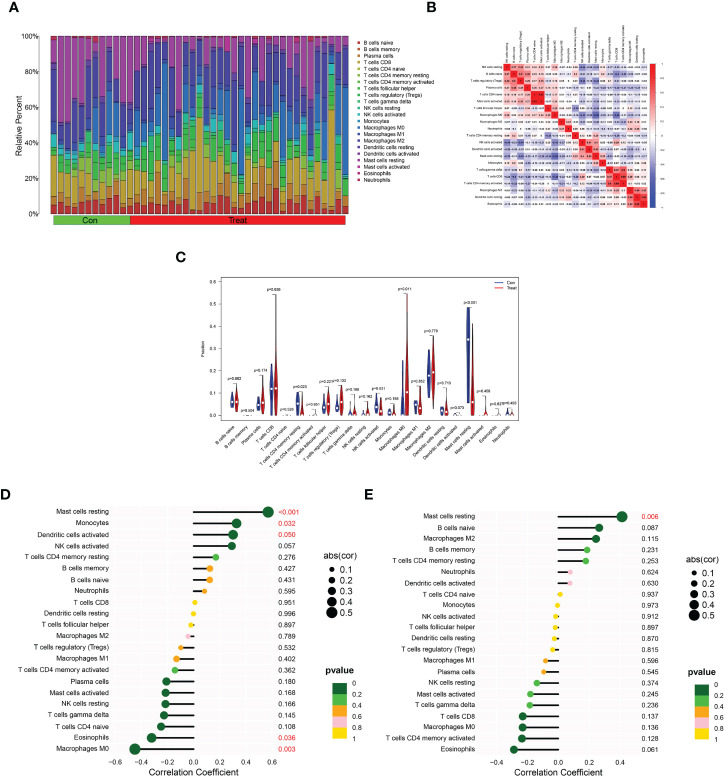
Distribution and visualization of immune cell infiltration and correlation analysis. **(A)** Barplot showing the proportion of 22 immune cell subtypes between ULMS and ULM samples. **(B)** Heatmap showing the correlation matrix of all 22 immune cell subtype compositions. Both horizontal and vertical axes demonstrate immune cell subtypes. Immune cell subtype compositions (higher, lower, and same correlation levels are displayed in red, blue, and white, respectively), and Pearson coefficient was used for significance test. **(C)** Violin plot showed the the total distribution of immune cells in ULMS and ULM samples. Correlation between *DPP6* **(D)**, *MFAP5* **(E)** and infiltrating immune cells in ULMS.

Next, we verified the correlation of the *DPP6* and *MFAP5* genes with immune cell infiltration. We determined the proportions of 22 immune cells in the ULMS and ULM samples using the CIBERSORT algorithm (**Figures 7A, B**). The components of immune cells in the ULMA vs. ULM group were explored. The ratios of resting CD4+ memory T cells (P =0.023), activated NK cells (P = 0.031) and resting mast cells (P <0.001) in the ULMS group were markedly lower than those in the ULM group. However, the ratio of M0 macrophages (P = 0.011) was significantly higher in ULMS compared to ULM (**Figure 7C**). Furthermore, we studied the relationship between the *DPP6* and *MFAP5* genes and infiltrating immune cells. *DPP6* was positively related to resting mast cells (r = 0.570, p < 0.001), monocytes (r = 0.328, P = 0.032) and activated dendritic cells (r = 0.301, p =0.0495) but negatively related to eosinophils (r = −0.321, P = 0.036) and M0 macrophages (r = -0.450, P =0.003) (**Figure 7D**). Moreover, *MFAP5* was positively related to resting mast cells (r = 0.413, p = 0.006) (**Figure 7E**). These findings supported that *DPP6* and *MFAP5* are related to immune activity.

## Discussion

ULMS is one of the most common subtypes in mesenchymal neoplasms, but research on ULMS is limited. Because the incidence rate is low, different clinical features and histopathological appearances result in a lack of molecular biomarkers, offering no superior treatment regimen (21). The biological behaviour of ULMS is difficult to predict. Although the tumor is often restricted to the uterus, recurrence and metastasis are highly common (22). An increasing number of studies have employed immune cells as a new bioinformatic approach to investigate the diagnosis and prognosis of various diseases, including gastric cancer (23), breast cancer (24) and osteosarcoma (25). However, there are few studies on the immune cell infiltration association with DEGs in ULMS. Thus, we focused* *on the identification of significant diagnostic DEGs for ULMS and determined the correlation of these DEGs with infiltrating immune cells in ULMS.

To our knowledge, our study is the first to apply multiple GEO datasets for knowledge mining* *using a machine* *learning* *approach in ULMS to identify significant diagnostic biomarkers related to immune cells. In the present study, which utilized the GSE36610, GSE64763, GSE9511 and GSE68295 datasets from the GEO database, 55 DEGs were identified by comparing ULMS and ULM samples. DO enrichment showed that the 55 DEGs were mainly related to solid malignant tumors and haematological malignancies. KEGG analysis and GSEA indicated that the DEGs were involved in regulating immune-related pathways and the cell cycle. Risinger et al. reported that defective postreplication mismatch repair resulting in microsatellite instability is present in considerable portions of sarcomas in gynecology (26). Similarly, mismatch repair (MMR) protein has been screened in uterine carcinosarcomas and leiomyosarcomas by immunohistochemical assays but has not been identified in other types of uterine mixed epithelial/mesenchymal or mesenchymal malignancies (27). Anderson et al. found that p53 expression may act as a prognostic biomarker for ULMS (28). Abnormal p53 staining (null or strong/diffuse) has been observed in ULMS with 70% sensitivity and 100% specificity against inflammatory myofibroblastic tumors (IMTs) and is related to genomic alterations (29). Relevant study has demonstrated that HTLV-1 infection correlates with the occurrence of ULMS. However, HTLV-1 has been thoroughly studied in adult T-cell leukaemia/lymphoma (ATL) (30–32), an aggressive CD4+ T-cell malignancy. HTLV-1 increases genomic instability by directly altering the expression of host genes; conversely, abnormal gene expression may influence the longevity of infected CD+4 T cell clones and profileration rate, allowing further mutations to accumulate and the host genome structure to vary, ultimately leading to malignant transformation (33). Because HTLV-1 mediates immune-related pathways, it possible* *that regulation of the immune response is strongly associated with the occurrence of ULMS.

We identified two diagnostic biomarkers based on integrating two machine-learning algorithms and diagnostic ability analysis, and we verified these markers using the GSE9511 and GSE68295 datasets. The dipeptidyl peptidase 6 (*DPP6*) gene encodes a single transmembrane peptidase without activity. Most likely, *DPP6* enhances its expression and regulates its gating feature by combining at the permeation and gating modules of the potassium channel (34). In breast cancer tissues, *DPP6* has low expression at the transcription and protein levels, and in breast cancer patients, low expression of *DPP6* indicates poor prognoses, suggesting that *DPP6* may serve as a tumour suppressor in tumour development (35), which agreed with our study. However, in surgically treated clear cell renal cell carcinoma (ccRCC) patients, the promoter methylation of *DPP6* genes is related to an aggressive phenotype and early progression of distant metastasis (36). Similarly, in pancreatic ductal adenocarcinoma tissues, the promoter methylation of *DPP6* genes is significantly higher than that in normal tissues (37). Microfibril-associated protein 5 (*MFAP5*) is a 25 kDa glycoprotein present in the extracellular matrix and stroma in all tissues (38), and it is crucial for elastic microfibril assembly. Using a microarray to investigate prostate tumors, researchers have detected 3800 significant expression alterations between the tumor stroma and benign stroma, and they reported that the downregulation of *MFAP5* expression is the most significant alteration in the prostate cancer stroma among all genes examined (39). Significant loss of *MFAP5* expression in colon cancer stroma may facilitate the difference between pseudoinvasive and true invasive tumors with a specificity of 75% and a sensitivity of 80% in colonic adenomatous polyps (40). However, high expression levels of *MFAP5* are associated with a worse prognosis in ovarian cancer (both in epithelium and stroma). In the present study, we observed significantly low expression of *MFAP5* in the stromal component of ULMS specimens, similar to the above study.

We applied CIBERSORT to assess the types of immune cell infiltration in ULMS and ULM. We discovered that decreased infiltration of resting CD4+ memory T cells, activated NK cells and resting mast cells in addition to increased infiltration of M0 macrophages were potentially correlated with the occurrence and development of ULMS. Xiaoqing et al. found that the infiltration of two types of immune cells (resting mast cells resting and activated NK cells) is lower in ULMS tissues, while the infiltration of five types of immune cells (memory B cells, M0 macrophages, activated mast cells, M1 macrophages and follicular helper T cells) is higher in ULMS tissues than in normal myometrium (NL) tissues (41). Similarly, our study demonstrated that the infiltration of immune cell types was lower due to the selection of the control group. Additionally, we found that the *DPP6* gene was positively correlated with resting mast cells, monocytes and activated dendritic cells. However, M0 macrophages and eosinophils had a negative correlation with the *DPP6* gene. Together, these findings indicated that the *DPP6* gene is associated with several types of immune cell infiltration and plays an important role in ULMS, suggesting that should* *be* *a focus* *in* *future* *experimental work.

The present study had limitations. First, due to the low incidence rate of ULMS, the number of cases was not enough in the GSE36610 and GSE64763 datasets. Second, the function and reproducibility of the *DPP6* and *MFAP5* genes as well as the related immune cell infiltration should be further validated by prospective studies with larger sample sizes in ULMS.

## Conclusion

Based on the GEO database, the two hub genes and the infiltration of five types of immune cells were related to ULMS occurrence. *DPP6* and *MFAP5* genes may affect the occurrence of ULMS through immune-related pathways. Thus, these findings provided molecular evidence for the treatment of ULMS in the future.

## Data availability statement

The original contributions presented in the study are included in the article/**Supplementary Material**. Further inquiries can be directed to the corresponding author.

## Ethics statement

The studies involving human participants were reviewed and approved by Ethics committee of The Second Affiliated Hospital of Fujian Medical University. The patients/participants provided their written informed consent to participate in this study.

## Author contributions

All authors contributed to the article and approved the submitted version.

## Funding

This work was supported by the Fujian Provincial Health Technology Project (No. 2019-1-15).

## Acknowledgments

The authors acknowledge the GEO database for providing data of ULMS and ULM available.

## Conflict of interest

The authors declare that the research was conducted in the absence of any commercial or financial relationships that could be construed as a potential conflict of interest.

## Publisher’s note

All claims expressed in this article are solely those of the authors and do not necessarily represent those of their affiliated organizations, or those of the publisher, the editors and the reviewers. Any product that may be evaluated in this article, or claim that may be made by its manufacturer, is not guaranteed or endorsed by the publisher.
