# Supplementary material for: DPP6 and MFAP5 are associated with immune infiltration as diagnostic biomarkers in distinguishing uterine leiomyosarcoma from leiomyoma
Source: Front Oncol. 2022 Nov 30;12:1084192. doi: 10.3389/fonc.2022.1084192 (PMC9748670; doi:10.3389/fonc.2022.1084192)
Supplement: Supplementary file 1 [file Table_1.docx]

Table S1: Enrichment analyses via gene set enrichment analysis in ULM group

| Enrichment Description | pvalue | p.adjust |
| --- | --- | --- |
| KEGG_cell adhesion molecules cams | 6.07E-05 | 0.0014 |
| KEGG_focal adhesion | 4.01E-07 | 2.44E-05 |
| KEGG_metabolism of xenobiotics by cytochrome P450 | 4.66E-06 | 0.0002 |
| KEGG_neuroactive ligand receptor interaction | 5.15E-05 | 0.0013 |
| KEGG_wnt signaling pathway | 2.15E-05 | 0.0008 |
