# Supplementary material for: DPP6 and MFAP5 are associated with immune infiltration as diagnostic biomarkers in distinguishing uterine leiomyosarcoma from leiomyoma
Source: Front Oncol. 2022 Nov 30;12:1084192. doi: 10.3389/fonc.2022.1084192 (PMC9748670; doi:10.3389/fonc.2022.1084192)
Supplement: Supplementary file 2 [file Table_2.docx]

Table S2: Enrichment analyses via gene set enrichment analysis in ULMS group

| Enrichment Description | pvalue | p.adjust |
| --- | --- | --- |
| KEGG_base excision repair | 0.0007 | 0.0090 |
| KEGG_cell cycle | 1.00E-10 | 9.10E-09 |
| KEGG_DNA replication | 1.00E-10 | 9.10E-09 |
| KEGG_mismatch repair | 5.12E-05 | 0.0013 |
| KEGG_oocyte meiosis | 0.0002 | 0.0031 |
